# Characterization of a new selective glucocorticoid receptor modulator with anorexigenic activity

**DOI:** 10.1038/s41598-024-58546-1

**Published:** 2024-04-03

**Authors:** Junekyoung Lee, Yeonghun Song, Young A. Kim, Intae Kim, Jooseon Cha, Su Won Lee, Yoonae Ko, Chong-Su Kim, Sanghee Kim, Seunghee Lee

**Affiliations:** 1 College of Pharmacy and Research Institute of Pharmaceutical Sciences, Seoul National University, Seoul, 08826 Korea; 2 Department of Food and Nutrition, College of Natural Information Sciences, Dongduk Women’s University, Seoul, 02748 Korea

**Keywords:** Molecular biology, Neuroscience, Endocrinology

## Abstract

Obesity, a worldwide epidemic, leads to various metabolic disorders threatening human health. In response to stress or fasting, glucocorticoid (GC) levels are elevated to promote food intake. This involves GC-induced expression of the orexigenic neuropeptides in agouti-related protein (AgRP) neurons of the hypothalamic arcuate nucleus (ARC) via the GC receptor (GR). Here, we report a selective GR modulator (SGRM) that suppresses GR-induced transcription of genes with non-classical glucocorticoid response elements (GREs) such as *Agrp*-GRE, but not with classical GREs, and via this way may serve as a novel anti-obesity agent. We have identified a novel SGRM, 2*-O-trans*-*p*-coumaroylalphitolic acid (Zj7), a triterpenoid extracted from the *Ziziphus jujube* plant, that selectively suppresses GR transcriptional activity in *Agrp*-GRE without affecting classical GREs. Zj7 reduces the expression of orexigenic genes in the ARC and exerts a significant anorexigenic effect with weight loss in both high fat diet-induced obese and genetically obese *db/db* mouse models. Transcriptome analysis showed that Zj7 represses the expression of a group of orexigenic genes including *Agrp* and *Npy* induced by the synthetic GR ligand dexamethasone (Dex) in the hypothalamus. Taken together, Zj7, as a selective GR modulator, showed beneficial metabolic activities, in part by suppressing GR activity in non-classical GREs in orexigenic genes. This study demonstrates that a potential anorexigenic molecule may allow GRE-specific inhibition of GR transcriptional activity, which is a promising approach for the treatment of metabolic disorders.

## Introduction

To maintain energy homeostasis in the body, it is necessary to sense the peripheral signals that reflect the amount of stored energy in the body and deliver the information to the central nervous system (CNS). The hypothalamus in the CNS, consisting of multiple nuclei, regulates homeostasis crucial for survival and reproduction. Particularly, the arcuate nucleus (ARC), containing various neurons that control energy balance, reproduction, and growth, plays key roles in sensing/processing peripheral cues due to its permeability and proximity to the peripheral bloodstream^1–3^. In particular, agouti-related protein (AgRP) neurons in the ARC produce the key orexigenic neuropeptides AgRP and neuropeptide Y (NPY) that increase food intake and reduce energy expenditure in response to hunger signals^4–6^.

Glucocorticoid (GC), via the GC receptor (GR) that belongs to the nuclear receptor superfamily of ligand-dependent transcription factors, regulates the expression of many distinct target genes in metabolism, inflammation, stress response, and development^7^. GC is one of the well-known peripheral orexigenic signals and accordingly, the level of GC is increased in fasting conditions, activating the expression of AgRP and NPY^8,9^. The nature of GC response depends on cell types and context-specific actions of GR and GR-interacting transcription factors through different types of glucocorticoid response elements (GREs). GCs are the most widely used anti-inflammatory drugs and obesity, one of the major adverse effects of GCs, is thought to be caused by aberrant activation of orexigenic GR targets^10^. GR has been shown to partner with lineage-determining factors to generate cell-type-specific GC responses^11,12^. GR binds cis-regulatory elements together with Activator protein 1 (AP-1) and Nuclear factor kappa-light-chain-enhancer of activated B cells (NF-κB) in macrophages^13,14^. E47 co-regulates GR targets and modulates hepatic GC action in the liver^15^. The transcription factor brain-specific homeobox factor (Bsx) co-operates with GR to induce orexigenic AgRP expression in AgRP neurons^16^. Therefore, targeting specific regulatory modules between GR and interacting factors may provide an opportunity to develop new approaches that mitigate the orexigenic effects of GC while retaining potent anti-inflammatory properties that can ensure cell type and locus specific gene regulation by GR.

**Figure 1:**
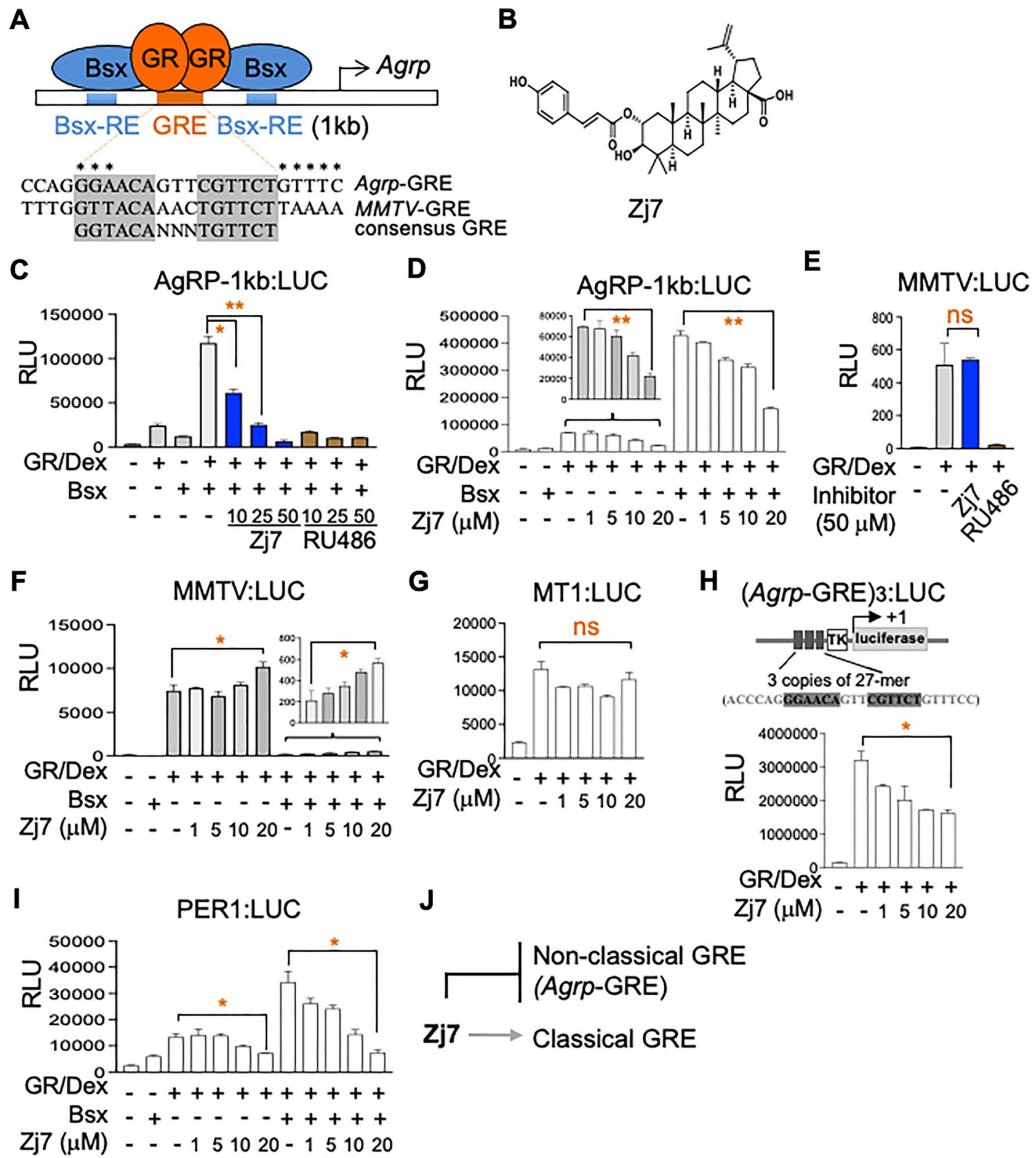
Selective inhibition of *Agrp*-GRE by a natural steroid compound. (**A**) Schematic representation of the *Agrp* promoter containing binding sites for Bsx and GR. *Agrp*-GRE, *MMTV*-GRE and consensus GRE are highlighted. Asterisks indicate the sequences in the first half site (GGA) and the region flanking the second half site (GTTTC) of *Agrp*-GRE that are critical for the unique properties of *Agrp*-GRE (i.e., synergy with Bsx in the induction of orexigenic genes). (**B**) Structure of the Zj7 compound. (**C**–**I**) Luciferase reporter assays with AgRP-1 kb:LUC, (*Agrp*-GRE)_3_:LUC, PER1:LUC, MMTV:LUC and MT1-LUC in HEK293T cells. AgRP-1 kb:LUC was synergistically activated by Bsx and GR treated with the synthetic GC, dexamethasone (Dex, 10^−7^ M). Treatment of naturally derived steroidal compound Zj7 suppressed AgRP-1 kb:LUC, (*Agrp*-GRE)_3_:LUC and PER1:LUC, but did not affect MMTV:LUC and MT1-LUC. RU486 inhibited both AgRP-1 kb:LUC and MMTV:LUC activated by GR and Dex. Transfections were repeated at least three times independently. Data are presented as the mean of triplicate values and error bars represent the standard deviation. Statistical differences were determined by Student’s t-test; *p < 0.05, and **p < 0.01 and ‘ns’ indicates not significant. (J) Zj7 negatively targets non-classical *Agrp*-GRE but not classical GRE.

Bsx is highly expressed in AgRP neurons and has been shown to link spontaneous locomotor activity for food seeking and food intake by regulating the expression of AgRP and NPY^17^. Previously, we have reported the mechanism by which GC triggers fasting-dependent induction of AgRP expression in AgRP neurons. This involves GR binding to the non-classical GRE in the *Agrp* gene in cooperation with Bsx bound to neighboring Bsx-binding motifs^16^. This unique *Agrp*-GRE mediates potent AgRP induction by the synergy of GR and Bsx, and we hypothesized that similar motifs are associated with other orexigenic genes such as *Period 1* (*Per1)* and *Ankyrin repeat and SOCS box containing 4* (*Asb4)* in AgRP neurons (Fig. 1A). Interestingly, Bsx rather inhibits transactivation directed by classical GREs, suggesting that it acts as a GRE dependent dual regulator of GC/GR target genes^16^.

We employed these biological findings to show the prospect of the identification of selective GR modulators (SGRMs) targeting genes with non-classical GREs. To identify compounds that selectively suppress the transcriptional activity of GR in non-classical *Agrp*-GRE but not in classical GREs, we first employed our similarity-implemented virtual screening approach and structure–activity relationship (SAR) analysis led us to identify 2-*O-trans-p*-coumaroylalphitolic acid (Zj7), also naturally derived from the plant *Ziziphus jujube* (Fig. 1B)^18,19^. Our study identifies Zj7 as an important anorexigenic molecule and proposes a potential molecular mechanism for the treatment of obesity.

## Results

### Selective inhibition of *Agrp*-GRE and similar GREs but not classical GREs by naturally derived steroidal compound

To test if the synergistic transcriptional activity of GR and Bsx is selectively blocked by Zj7, we adopted the cell-based assay using luciferase reporters. AgRP-1 kb:LUC reporter was constructed by linking the 1-kb *Agrp* promoter containing two Bsx-REs and GRE to luciferase gene as described previously (Fig. 1A). Interestingly, Zj7 suppressed the transcriptional activity of AgRP-1 kb:LUC driven by dexamethasone (Dex) in HEK293T cells transfected with the expression vectors for GR and Bsx (Fig. 1C and D). Furthermore, Zj7 showed no effect or even increased the Dex/GR-dependent activity of mouse mammary tumor virus (MMTV):LUC (Fig. 1E and F) and another GR target gene reporter containing classical GRE, Metallothionein 1 (MT1):LUC (Fig. 1G), suggesting that Zj7 does not simply block the ligand-bound GR activity. In stark contrast, RU486, an antagonist of GR, strongly inhibited both AgRP-1 kb:LUC and MMTV:LUC (Fig. 1C and E).

To further examine the detailed molecular basis of Zj7 in inhibiting AgRP-1 kb:LUC, we treated Zj7 with or without Bsx expression and found that Zj7 suppressed GR transcriptional activity in a dose dependent manner in both conditions. Even though the activation of AgRP-1 kb:LUC by Dex/GR alone was much lower than the synergistic activation by Dex/GR and Bsx, it was still significantly suppressed by Zj7 (Fig. 1D). Dex response of (*Agrp*-GRE)_3_:LUC with three copies of 27-mer sequence containing *Agrp*-GRE alone was also inhibited by Zj7 (Fig. 1H), suggesting that the selective repression of AgRP-1 kb by Zj7 can be mediated by the unique nature of *Agrp*-GRE alone. To test whether Zj7 affects the activation of other GR target genes directed by GREs similar to *Agrp*-GRE, we used PER1:LUC. *Per1*-GRE shares the characteristic features of *Agrp*-GRE, sequences in the first half site (GGA) and the flanking region of the second half site (GTTTC) (Fig. 1A, asterisks and Supplementary Figure S1). The reporter was activated by Dex/GR alone and synergistically by Dex/GR and Bsx, and these activations both were blunted by Zj7 treatment (Fig. 1I). Overall, these results suggest that Zj7 selectively antagonizes the action of GC in non-classical GRE such as *Agrp*-GRE but not in classical GREs such as *MMTV*-GRE and *MT1*-GRE (Fig. 1J). We speculate that the inhibitory action of Zj7 is possibly mediated by the suppression of the cooperative activity of the ligand-bound GR and Bsx as well as by the unique sequence features of the *Agrp*-GRE sequences.

### Inhibition of GR/Bsx synergistic transactivation by Zj7: modulation of GR-Bsx interaction and disruption of GR recruitment to target loci in the hypothalamus

**Figure 2:**
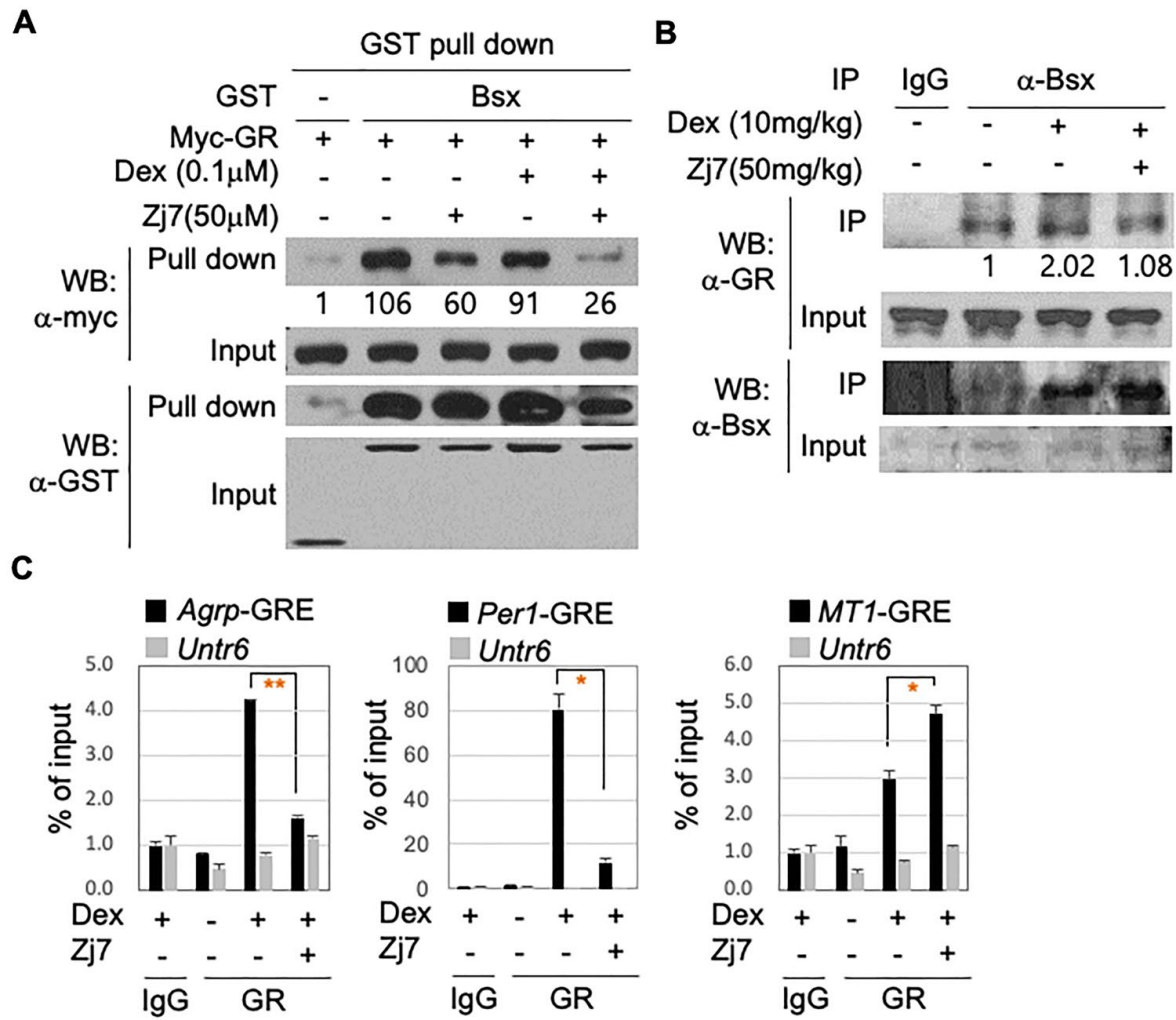
Zj7 modulates the GR activity by weakening the interaction with Bsx and interfering the recruitment of GR to *Agrp*-GRE (**A**) In vivo GST pull-down assays in HEK293T cells expressing GST-Bsx and Myc-GR. Zj7 treatment blocks the interaction between Bsx and GR. (**B**) CoIP analysis using hypothalamus tissue from ten-week-old male mice treated with either vehicle, Dex (10 mg/kg), or Dex (10 mg/kg) + Zj7 (50 mg/kg) for 2 h. (**C**) ChIP assays with mHypoA-2/12 cells treated with vehicle, Dex (0.1 μM) and Zj7 (50 μM) for 2 h. The recruitment of GR by Dex to *Agrp*-GRE and *Per1*-GRE was abolished by Zj7 treatment, while the recruitment of GR to *MT1*-GRE was further increased by Zj7. Data are mean ± standard error of the mean (SEM). Statistical differences were determined by Student’s t-test; *p < 0.05, and **p < 0.01.

To understand the molecular basis of the inhibitory action of Zj7 on synergistic GR/Bsx transactivation, we tested whether the interaction between GR and Bsx was inhibited by Zj7. We performed in vivo glutathione S-transferase (GST) pull-down assays using the lysates of HEK293T cells transfected with GST-fused Bsx and Myc-tagged GR. GR and Bsx interacted with each other, which was blocked by Zj7 treatment (lanes 3 and 5 in Fig. 2A).

Next, to examine whether the interaction of GR and Bsx expressed endogenously in the hypothalamic tissues was also affected by Zj7, we used cell lysates of the mouse hypothalamus injected with the vehicle, Dex, or Dex plus Zj7 for two hours, then pulled them down using an anti-Bsx antibody, followed by western blotting with an anti-GR antibody. We observed the interaction of endogenous GR and Bsx, which was further enhanced by Dex treatment and attenuated by Zj7 treatment in the hypothalamic tissues (Fig. 2B).

Given the negative regulation of Zj7 towards non-classical *Agrp*-GRE without affecting classical GREs, we tested whether Zj7 recognizes GR bound to unique DNA sequences in *Agrp*-GRE and similar GREs and prevents GR recruitment to target loci. We performed a chromatin immunoprecipitation (ChIP) assay with either control immunoglobulin G (IgG) or an anti-GR antibody using the murine hypothalamic cell line HypoA-2/12 treated with the vehicle, Dex, or Dex plus Zj7. As expected, we found that GR was recruited to the GR target gene loci in a Dex-dependent manner, but not to the control region of the *untr6* locus. Surprisingly, the recruitment of GR to the *Agrp*-GRE was significantly downregulated by Zj7. Similar results were obtained for *Per1*-GRE, while the binding of GR to the *MT1*-GRE, which contains a classical GRE, was further enhanced by Zj7 (Fig. 2C). These results suggest that Zj7 targets the transcriptional activity of GR associated with *Agrp*-GRE, possibly by modulating the interaction between GR and Bsx, and by blocking the binding of GR to the distinct DNA sequences in *Agrp*-GRE, which likely underlies the selectivity of Zj7 towards some hypothalamic metabolic homeostasis genes regulated by glucocorticoids.

### Suppression of fasting-induced expression of *Agrp* and *Npy* in mouse hypothalamic ARC by acute Zj7 treatment

Based on the selective mode of action of Zj7 in suppressing GR transcriptional activity in *Agrp*-GRE, we tested whether Zj7 inhibits fasting-induced expression of AgRP in the ARC. We injected wild-type (WT) mice intraperitoneally with either vehicle or Zj7 (50 mg/kg) and subjected the mice to fasting for twelve hours. In situ hybridization (ISH) analysis showed that Zj7 significantly suppressed the fasting-mediated induction of *Agrp* and *Npy* compared to vehicle injection (Supplementary Figure S2). Immunohistochemistry (IHC) analysis of Orthopedia homeobox (OTP), a marker of AgRP neurons, showed that it was not affected by Zj7, suggesting that Zj7 selectively inhibits the expression of the orexigenic genes *Agrp* and *Npy* without affecting the survival of AgRP neurons (Supplementary Figure S2). To further investigate whether Zj7 caused any general adverse effects, we performed the elevated plus maze test and the conditioned taste aversion memory test and found that Zj7 did not cause any abnormal behavior such as anxiety (Supplementary Figure S3). Despite the Zj7 injections, no aversive behavior was observed for the consumption of 0.1% sucrose water in the conditioned taste aversion test. Interestingly, both the vehicle-injected and the Zj7-injected groups showed a substantial increase in sucrose consumption during retrieval, reaching approximately 116% and 186%, respectively. Moreover, sucrose consumption increased significantly with Zj7 injection. The results suggest that Zj7 may have an unexpected effect by increasing, rather than decreasing, sucrose consumption. This suggests a significant influence on retrieval processes and the potential modulation of taste-related behaviors, which may indicate a reduction in depression-like symptoms.

### Amelioration of obesity by Zj7 in high fat diet-induced obese mice

**Figure 3:**
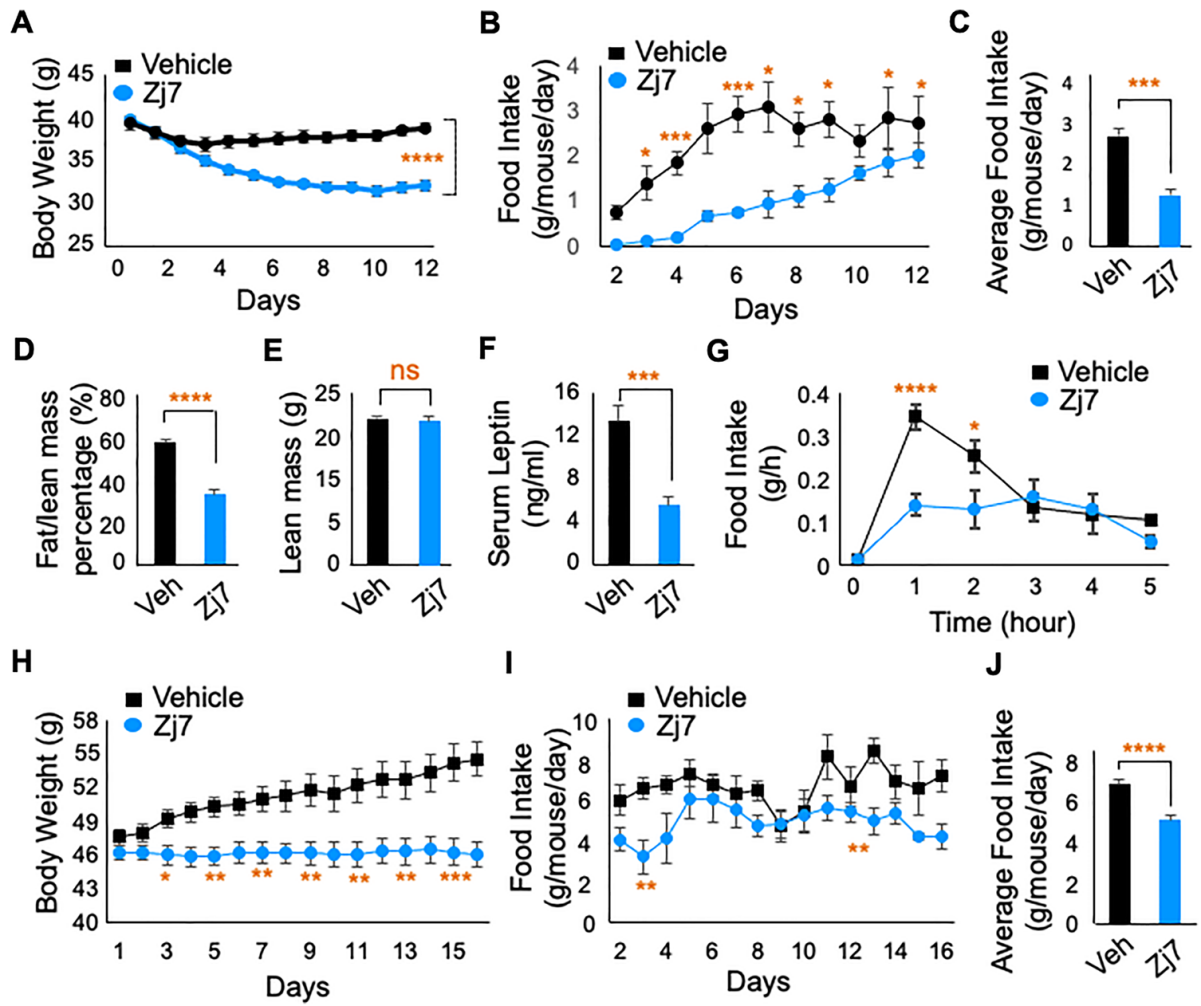
Anti-obesity effect of Zj7 on high fat diet induced obese (DIO) mice and *db/db* mice. Twelve-week-HFD-fed obese mice (**A**–**G**) were daily treated with vehicle or Zj7 (50 mg/kg) intraperitoneally (i.p.) for two weeks. (**A**) Body weight (g) (n = 10 for each group) and (**B**) Daily food intake of DIO mice for 12 days. (**C**) Average food consumption of DIO mice during 5–12 days of treatment. (**D**) Fat content normalized by lean mass (%) and (**E**) Lean mass (g) of DIO mice after two weeks of treatment (n = 10 for each group). (**F**) Serum leptin levels of vehicle (n = 10) or Zj7 (n = 8; 50 mg/kg) treated DIO mice after two weeks treatment. (**G**) Twelve-week-HFD-fed obese mice were fasted for sixteen hours then, treated with vehicle or Zj7 (50 mg/kg) i.p. and fed with food. Food intake after refed (g). (**H**–**J**) Six-week-old male *db/db* mice were treated with vehicle or Zj7 (50 mg/kg) for sixteen days (daily, i.p). (**H**) Body weight (g). **(I**) Daily food intake of *db/db* mice for 16 days. (**J**) Average food consumption of *db/db* mice during the treatment. (vehicle n = 9, Zj7 n = 10). Data are mean ± SEM. Statistical differences were determined by Student’s t-test; *p < 0.05, **p < 0.01, ***p < 0.001, ****p < 0.0001, and ‘ns’ indicates not significant.

Based on the inhibitory action of Zj7 on the expression of the orexigenic neuropeptides AgRP and NPY, we hypothesized that Zj7 reduces food intake, resulting in improved overnutrition-induced weight gain. To test this hypothesis, we intraperitoneally (i.p) injected high fat diet-induced obese (DIO) mice (mice fed high fat diet for 12 weeks) with either the vehicle or Zj7 for two weeks. While the body weight of vehicle-treated DIO mice showed virtually no change, the administration of Zj7 markedly reduced the body weight of DIO mice, from 39.74 ± 1.59 g to 32.14 ± 1.86 g (on day 1 versus day 12 of treatment, *p* < 0.0001), which is equivalent to 18.8 ± 3.78% weight loss relative to their initial weights (Fig. 3A). During the study, the daily food intake of Zj7-treated mice was significantly reduced compared to that of control mice (Fig. 3B). However, the data itself showed that Zj7-treated mice appeared to stop eating for 4 days, but these mice looked and behaved almost normal. We suspect that the first 4 days of feeding data were due to a technical error in measuring high fat diet (HFD) intake. The average of cumulative food consumption was calculated excluding the first 4 days of the study, which shows a significant difference in food intake between the vehicle and Zj7-injected groups (Fig. 3C). Our magnetic resonance imaging (MRI) results also revealed that the percentage of fat mass normalized by lean mass was reduced, while lean mass was not affected in Zj7-treated mice compared to vehicle-treated control mice (Fig. 3D and E). Serum leptin levels also decreased in Zj7-treated mice, reflecting reduced fat mass (Fig. 3F).

Next, to test whether Zj7 could suppress the hyperphagic behavior due to the reduced expression of the orexigenic neuropeptides AgRP and NPY induced by fasting, DIO mice were fasted for sixteen hours, then treated with vehicle or Zj7 and re-fed. Food intake was measured for the next five hours after refeeding. Vehicle-treated mice showed a dramatic increase in food intake after refeeding 1–2 h later, while Zj7-treated mice showed a significant decrease in food intake compared to vehicle-treated mice (Fig. 3G).

### Anti-obesity effect of Zj7 in *db/db* mice

To further test the effect of Zj7 in improving metabolic homeostasis in a genetically induced obese mouse model, we investigated whether Zj7 can reverse or block the progression of obesity in *db/db* mice, which carry elevated levels of leptin but are leptin-resistant due to a mutation in the gene encoding the leptin receptor. Vehicle-treated *db/db* mice gained weight during the experimental period. At the end of the experiment, the *db/db* mice had gained 13% of their initial body weight. Surprisingly, administration of Zj7 to the *db/db* mice prevented the weight gain and they maintained their initial body weight throughout the experiment (Fig. 3H). We observed a significantly lower food intake in Zj7-treated *db/db* mice than in vehicle-treated *db/db* mice (Fig. 3I and J), which partially explains how Zj7 prevents further weight gain in *db/db* mice.

### Enhanced glucose and energy homeostasis by Zj7 in DIO mice

**Figure 4:**
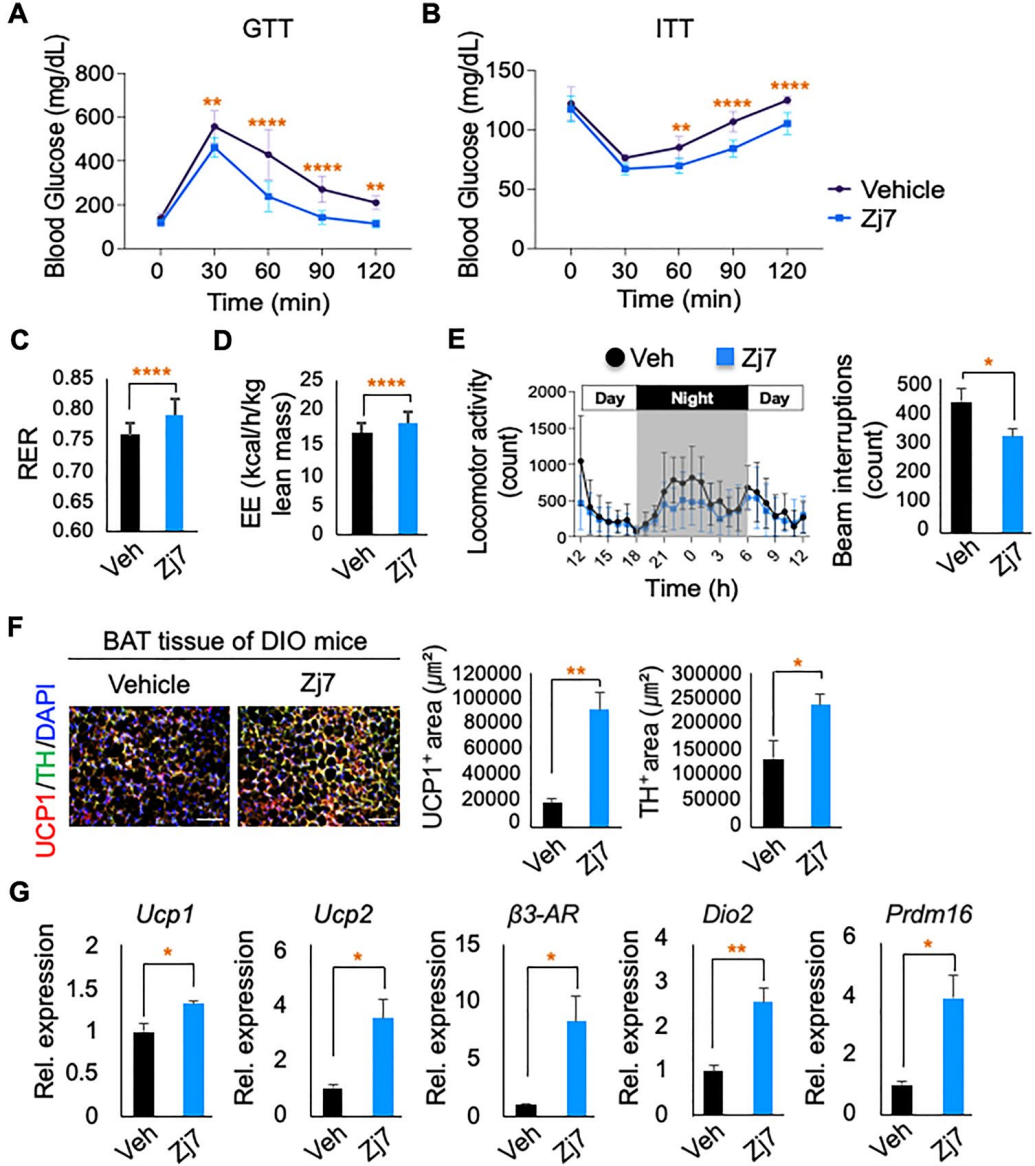
Improvement of glucose and energy homeostasis by Zj7 treatment. (**A**) Glucose tolerance test (GTT) after two-week treatments. (**B**) Insulin tolerance test (ITT) after 17-day treatments. (Vehicle n = 8; Zj7 n = 8). (**C**) RER. (**D**) EE (kcal/h/kg lean mass). (**E**) Locomotor activity (count). Bar graphs represent the average of 48 hourly observations for each of the vehicle-injected and Zj7-injected groups. (Vehicle n = 10, Zj7 n = 9). (**F**) IHC showed that brown adipose tissue from Zj7 injected DIO mice significantly increased the expression of UCP1 (thermogenic gene) and TH (sympathetic innervation) levels compared to vehicle injected DIO mice. (Vehicle n = 3, Zj7 n = 3). Scale bars: 100 μm. (**G**) The thermogenesis markers, including *Ucp1, Ucp2, β3-AR, Dio2* and *Prdm16* were significantly increased in brown adipose tissue of Zj7 injected DIO mice compared to vehicle injected DIO mice by quantitative RT-PCR (Vehicle n = 3, Zj7 n = 3). The values are presented as the means ± SEM. *p* values were calculated using Bonferroni’s post hoc test following a two-way analysis of variance (ANOVA) (A and B) using Prism 9 (GraphPad) software and Student’s t-test (**C**–**G**). The number of asterisks indicates the level of significance: *p < 0.05, **p < 0.01, ***p < 0.001 and ****p < 0.0001.

To test whether Zj7 can improve glucose homeostasis in DIO mice, we performed a glucose tolerance test (GTT) following treatment of DIO mice with vehicle or Zj7 for two weeks. Blood glucose level decreased more rapidly in the Zj7-treated mice than in the vehicle-treated mice (Fig. 4A). Seventeen days of Zj7 treatment also enhanced insulin sensitivity in DIO mice, as analyzed using an insulin tolerance test (ITT) (Fig. 4B).

In addition to its inhibitory effects on food intake and body weight gain, we reasoned that Zj7 would also facilitate energy expenditure, given the well-known inhibitory action of AgRP neurons on energy expenditure. To test whether Zj7 enhances energy expenditure, we measured various metabolic parameters (respiratory exchange ratio [RER], energy expenditure [EE]) in vehicle- or Zj7-treated DIO mice. The RER of the Zj7-treated mice was higher than that of the vehicle-treated mice (Fig. 4C). In addition, the EE levels normalized to the lean mass were significantly increased in Zj7-treated DIO mice (Fig. 4D). To further test whether the increased energy expenditure was due to an increase in locomotor activity, we analyzed the locomotor activity with beam interruption counts. Interestingly, Zj7-injected DIO mice showed even lower activity compared to vehicle-injected DIO mice (Fig. 4E).

Next, we analyzed resting energy expenditure by examining whether thermogenic energy expenditure was increased. IHC for thermogenic markers in brown adipose tissue (BAT) showed that uncoupling protein-1 (UCP1) levels were significantly increased and tyrosine hydroxylase (TH) expression was also increased in Zj7-injected DIO mice, indicating that the sympathetic innervation was significantly enhanced (Fig. 4F). We also examined the expression of thermogenic genes such as *Ucp1, Ucp2, β3-AR, Dio2* and *Prdm16* in BAT, which were significantly upregulated in Zj7-injected DIO mice by quantitative reverse transcription PCR (qRT-PCR) (Fig. 4G). Overall, these results demonstrate that Zj7 increases energy expenditure mainly by enhancing resting energy expenditure.

### Changes of Dex-regulated gene expression by Zj7 in mouse hypothalamus

**Figure 5:**
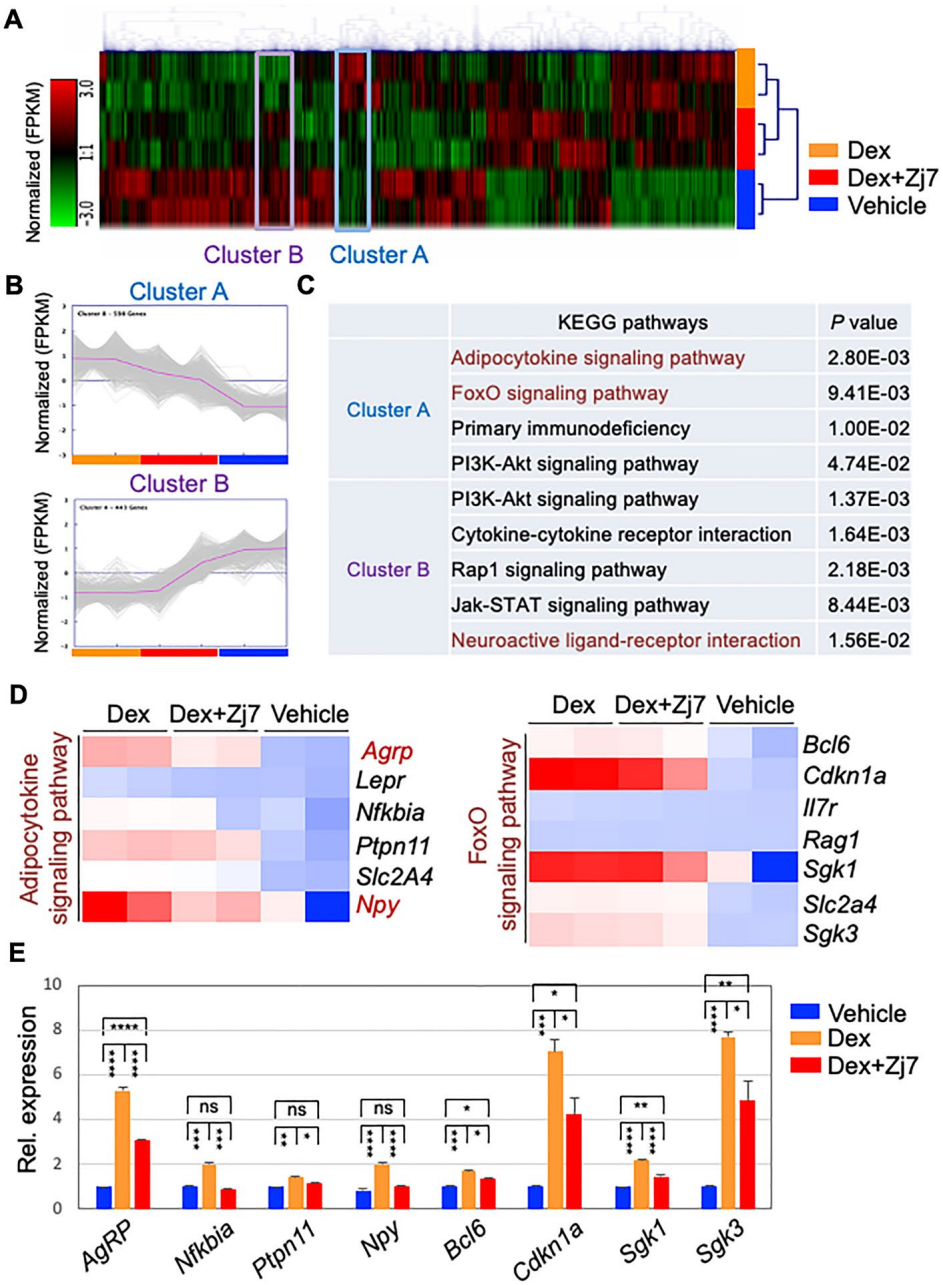
Zj7 modulates the expression of genes in mouse hypothalamus. (**A**–**E**) Transcriptome analysis of hypothalamic ARC tissue from 10-week-old male C57BL/6 mice injected i.p. for 24 h with vehicle, Dex (10 mg/kg) or Dex (10 mg/kg) + Zj7 (50 mg/kg). (**A**) Hierarchical clustering analysis shows the gene sets that were significantly altered by Dex and Zj7 treatment (log2 fold change 1.5, *p* < 0.05). Colors in the heatmap indicate z-scored expression values: red and green color indicate up- and down-regulated expressions, respectively. (**B**) Cluster A (N = 598) contains genes that are upregulated by Dex relative to vehicle treatment and then downregulated by Zj7, while Cluster B (N = 443) contains genes that are downregulated by Dex relative to vehicle treatment and then upregulated by Zj7. (**C**) KEGG pathway analysis with significant *p* values for cluster A and cluster B. (**D**) Heatmap shows that Dex-dependent gene expression in the adipocytokine signaling pathway and FoxO signaling pathway is repressed by Zj7. Colors in the heatmap display z-scored expression values: red and blue color indicate up- and down-regulated expressions, respectively. (**E**) The expression levels of selected genes in the hypothalamic ARC are significantly altered by Dex and Zj7, as determined by quantitative RT-PCR analysis. Error bars represent the SD of the mean. Statistical difference was determined by one-way ANOVA followed by Turkey’s test using Prism 9 (GraphPad) software; *p < 0.05, **p < 0.01, ***p < 0.001, ****p < 0.0001, and ‘ns’ indicates not significant.

Given the inhibitory actions of Zj7 on GR transactivation and weight gain, we hypothesized that in addition to AgRP and NPY, Zj7 would regulate the expression of genes related to food intake and/or energy homeostasis induced by GR activation. To identify genes regulated by Zj7 in the ARC of hypothalamus, we performed RNA sequencing (RNA-seq) experiments on hypothalamic ARC tissue from mice treated with vehicle, Dex and Dex plus Zj7 for twenty-four hours. We first identified 3151 differentially expressed genes (DEGs) with a log_2_ fold change (FC) ≥ 1.5 (*p* < 0.05), indicating a significant difference in gene expression in each condition (Fig. 5A). Hierarchical clustering analysis was conducted to identify gene sets that were differentially regulated by each condition (Fig. 5A). It is interesting to note that there were gene sets that were upregulated by Dex compared to vehicle and then downregulated by Zj7 treatment, that we classified as cluster A (N = 598). Cluster B (N = 443) contains genes that were downregulated by Dex compared to vehicle and then recovered by Zj7 (Fig. 5A and B). We further performed functional analysis for those DEGs in each cluster, and found that cluster A contains genes involved in the adipocytokine signaling pathway, and Forkhead box O (FoxO) signaling pathway, while cluster B contains genes involved in the phosphatidylinositol-3-kinase (PI3K)-Akt signaling pathway, Janus kinase (JAK)-signal transducer and activator of transcription (STAT) signaling pathway, and neuroactive ligand-receptor interaction, which are widely involved in metabolic homeostasis (Fig. 5C). As expected, we found that *Agrp* and *Npy* were upregulated by Dex and then repressed by Zj7 treatment (Fig. 5D). These genes identified in RNA-seq were independently validated (Fig. 5E).

### Downregulated expression of the orexigenic neuropeptides in hypothalamic ARC of Zj7- treated DIO mice

**Figure 6:**
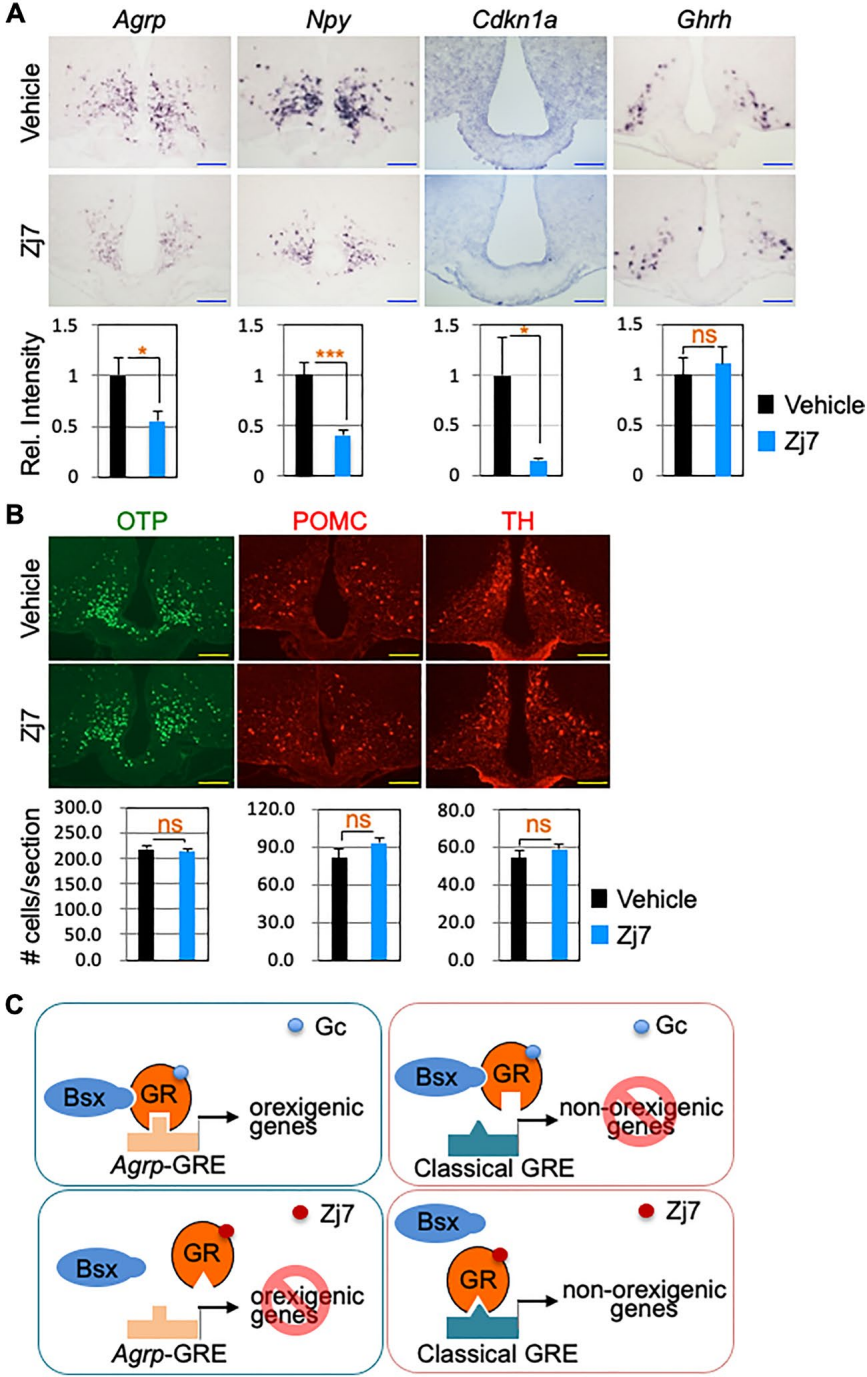
Suppression of orexigenic gene expression by Zj7 in hypothalamic ARC of DIO mice. Twelve-week-HFD-fed obese mice were subjected to a two-week treatment with the vehicle or Zj7 (50 mg/kg) (daily, i.p.). (**A**) In situ hybridization (ISH) results showed that the expression of *Agrp*, *Npy* and *Cdkn1a,* but not *Ghrh*, is significantly downregulated by Zj7 treatment (vehicle, n = 7; Zj7, n = 6). (**B**) Immunofluorescence images of OTP, POMC and TH (vehicle, n = 7; Zj7, n = 6). Representative images are shown. Quantification was based on the relative intensity of ISH signals or by counting the number of labelled cells using ImageJ in three rostral to caudal sections for each mouse. Data are expressed as mean ± SEM. Statistical differences were determined by Student’s t-test; *p < 0.05, ***p < 0.001, and ‘ns’ indicates not significant. Scale bars: 100 μm. (**C**) The working model for the selective action of Zj7. Zj7 impinges on the orexigenic action of GR by modulating the association of GR and Bsx and preventing the recruitment of GR to *Agrp*-GRE but not to classical GRE.

To test whether one of the mechanisms of the reduced food intake and weight loss in Zj7-treated DIO mice was the result of downregulated expression of orexigenic neuropeptides in hypothalamic ARC of Zj7-treated DIO mice, we examined the expression of *Agrp* and *Npy* by ISH. We also examined whether the expression of *Cdkn1a* was affected. The expression of *Agrp, Npy and Cdkn1a* in the ARC was markedly repressed in Zj7-treated mice compared to vehicle-treated mice, whereas the expression of the growth hormone-releasing hormone (*Ghrh*) in the ARC was not affected (Fig. 6A). IHC analysis showed that the numbers of OTP^+^-, pro-opiomelanocortin (POMC)^+^- and TH^+^-neurons were unaffected by Zj7 injection, suggesting that there were no issues with neuronal survival in the ARC (Fig. 6B).

## Discussion

In this report, we set out to identify small molecules that selectively suppress the transcriptional activity of GC/GR/Bsx in *Agrp*-GRE but not in classical GREs. Zj7, a novel natural steroidal compound, shows a significant and selective inhibition of *Agrp*-GRE transactivation without affecting the well-characterized classical GRE associated with either the promoter in *MMTV* or *MT1* gene. In DIO animal models, Zj7 suppresses food intake and increases energy expenditure, resulting in significant weight loss. RNA-seq analysis revealed that Zj7 represses the expression of a group of orexigenic genes induced by the synthetic GR ligand, Dex, in the hypothalamic ARC. Overall, our study shows that SGRMs that affect the activities of *Agrp*-GRE and similar GREs associated with other orexigenic genes provide a potential therapeutic approach to treat obesity.

Importantly, we provide the molecular basis for how Zj7 selectively regulates GR targets. Zj7 affects the expression of the orexigenic neuropeptides *Agrp*, *Npy* and other metabolic GR target genes, at least in part by targeting the unique nature of the *Agrp*-GRE sequence and the interactions between GR and Bsx. Our model suggests that Bsx interacts with GC-bound GR that recognizes *Agrp*-GRE in orexigenic genes, whereas this complex is unable to bind classical GRE in non-orexigenic genes. Zj7 selectively targets GR bound to the *Agrp*-GRE sequence, blocking the interaction between Bsx and GR and altering the structure of GR so that it does not recognize *Agrp*-GRE in orexigenic genes, but still occupies the classical GRE in non-orexigenic genes (Fig. 6C). These results further support our previous report indicating the importance of Bsx and *Agrp*-GRE in the induction of *Agrp* expression, and also lead to the discovery of a new SGRM acting on the orexigenic action of GR.

In particular, we have characterized the selectivity of Zj7, and luciferase reporter assays clearly show that Zj7 selectively antagonizes the action of GC in *Agrp*-GRE, but not in other classical GREs such as *MMTV*-GRE or *MT1*-GRE, whereas the classical GR antagonist RU486 inhibits both *Agrp*-GRE and classical GRE in *MMTV*, as expected (Fig. 1C–J). As for the unique mode of action of Zj7 in GR regulation, distinct from that of Dex or RU486, we have shown that Zj7 inhibits the interaction between Bsx and GR in hypothalamic tissues as well as in HEK293T cells overexpressing GR and Bsx (Fig. 2A and B). These results suggest that Zj7 may bind to the specific binding surface between GR and Bsx rather than to the ligand-binding domain of GR. However, Zj7 also suppressed the transcriptional activity of GR in *Agrp*-GRE without Bsx, raising another possibility for the action of Zj7 on the regulation of GR activity. Interestingly, the recruitment of GR by Dex to *Agrp*-GRE and *Per1*-GRE was significantly downregulated by Zj7, while the binding of GR to *MT1*-GRE was even enhanced by Zj7 (Fig. 2C). This result suggests the involvement of the unique GRE sequence itself in *Agrp*-GRE, which may induce the structural change of GR that is negatively affected by Zj7. To fully understand the molecular mechanism of how Zj7 regulates the GR activity in different GREs, further studies such as binding assays and molecular dynamic simulation analysis are required.

Primary reduction of food intake and consequent body weight loss of Zj7-treated DIO mice resulted in decreased fat content and restored leptin levels to normal (Fig. 3). More interestingly, Zj7 treatment in DIO mice fasted for sixteen hours suppressed the compulsive eating behavior after re-feeding (Fig. 3G), indicating that Zj7 has a potent anorexigenic effect. Consistent with our results, *Bsx* mutant mice showed reduced rebound hyperphagia after fasting^17^. In addition, Zj7 also improves glucose homeostasis in DIO mice (Fig. 4A and B). One definite molecular mechanism of how Zj7 exerts its anorexigenic action in DIO mice or *db/db* mice may be attributed to its suppressive effect on the expression of AgRP, NPY and other orexigenic genes and possibly FoxO signaling pathway genes (Figs. 5 and 6). It is interesting to note that there are different responses in food intake and body weight observed between DIO mice injected with Zj7, compared to *db/db* mice, which can be attributed to genetic factor. *Db/db* mice have high levels of leptin but are leptin resistant due to mutations in the gene encoding the leptin receptor, and exhibit metabolic problems including low body temperature at environmental temperatures and marked tolerance to cold^20,21^. We found that DIO mice treated with Zj7 increased thermogenic energy expenditure, suggesting that Zj7 potentiates leptin action in DIO mice.

Our systematic genome-wide analysis of transcriptional changes with or without Zj7 treatment in the mouse hypothalamus provided more putative target genes of GR to induce obesity. It is interesting to note that some genes, such as cyclin-dependent kinase inhibitor p21 (*Cdkn1a*), serum/glucocorticoid regulated kinase 1 and 3 (*Sgk1* and *Sgk3*) have previously been shown to be upregulated by stress hormones, corticosterone, and food restriction^22^. To investigate whether the genes identified from our RNA-seq could be direct targets of GR and have GREs similar to that of *Agrp*-GRE, we examined the reported chromatin immunoprecipitation sequencing (ChIP-seq) peaks for GR in mouse mammary adenocarcinoma cells^23^, as genome-wide ChIP-seq results for GR in hypothalamus have not yet been reported. Among the independently validated genes by qRT-PCR (Fig. 5E), *Cdkn1a, Sgk1, Nfkbia* and *Bcl6* had ChIP-seq peaks for GR. Strikingly, we found that *Cdkn1a* had a GRE similar to *Agrp*-GRE (Supplementary Figure S1). These results suggest that there are additional putative common target genes of GR and Bsx that contain the unique feature of *Agrp*-GRE, possibly in the orexigenic pathway, and thus Zj7 may have a broader impact in controlling energy balance.

Many centrally acting anti-obesity drugs suppress appetite, although their exact mechanisms of action are not well characterized. However, unwanted side effects limit their use as anti-obesity drugs^24,25^. This problem extends to GR antagonists, although their anti-obesity effects are relatively well documented. For example, the GR antagonist RU486 has been shown to reduce body weight gain and fat mass in *db/db* mice or *fa/fa* Zucker rats^26–31^. Recently, RU486, together with a PTP1b inhibitor, has been shown to promote weight loss and improve glucose homeostasis in obese mice^32^. CORT125281, a selective GR antagonist, also reduced body weight gain and fat mass, but was found to increase liver lipid accumulation^33^. GR antagonism with RU486 has favorable metabolic effects on body weight and fat mass, but is likely to cause side effects by inhibiting PR and AR transcriptional activity, while the use of CORT125281 may be limited due to adverse steatosis–inducing effects in mice. The observed increase in sucrose consumption despite the administration of the anorexigenic molecule Zj7 raises intriguing questions and warrants careful consideration. The effects of Zj7 on sucrose consumption may be context-dependent. For example, it could affect retrieval processes or alter the perception of the conditioned taste aversion test specifically, rather than exerting a general effect on appetite suppression. Further experiments, including detailed molecular and neural pathway analyses, may be required to understand the seemingly paradoxical effect of Zj7 on sucrose consumption. In addition, exploring the temporal dynamics of Zj7 effects and its specificity in different behavioral paradigms may contribute to a more comprehensive understanding of its impact on food intake behaviors. Further studies dissecting the interaction interfaces between Bsx and GR bound to *Agrp*-GRE and elucidating the structure of GR bound to Zj7 would facilitate our understanding of how Zj7 modulates the activity of GR in orexigenic gene expression and acts differently from other GR modulators.

In summary, by combining biochemical analysis, pharmacological approaches, and mouse models, we have demonstrated that Zj7 has a potent anti-obesity effect via its ability to suppress GR transcriptional activity in inducing *Agrp, Npy* and other orexigenic gene expression and to reduce feeding and body weight. If Zj7 shows anti-obesity activity in humans as it does in mouse models, targeting the selective GR action is likely to be a very promising strategy for the treatment of metabolic disorders.

## Materials and methods

### Luciferase reporter assays

HEK293T cells were maintained in Dulbecco’s modified essential medium (DMEM) supplemented with 10% fetal bovine serum (FBS) and penicillin/streptomycin. Cells were seeded into 48-well plates and incubated for 24 h, and transient transfections were performed with SuperFect (#301305; Qiagen). An actin promoter β-galactosidase plasmid was cotransfected to normalize the transfection efficiency. Zj7 (10–50 μM), RU486 (10–50 μM) and Dexamethasone (Dex) (100 nM) were treated for 18–20 h with DMEM supplemented with 10% charcoal stripped FBS. Cell extracts were analyzed for luciferase activity and values were normalized with β-galactosidase activity. Data are presented as means of duplicate values from represented experiments. Luciferase reporter assays were independently repeated at least three times. Data were shown in relative luciferase units (mean ± SD). The plant collection and use were in accordance with all the relevant guidelines.

### Mouse work

All mice were maintained on a normal 12 h light, 12 h dark cycle with ad libitum access to food and water, unless otherwise specified. For acute test of Zj7, 10-week-old C57BL/6 male mice were injected intraperitoneally (i.p) with Zj7 (50 mg/kg) or vehicle (corn oil) then fasted for 12 h. For test of Zj7 in DIO mice, 10-week-old C57BL/6 mice were fed high fat diet (TD.88137 42% from fat HFD; Envigo) for 12 weeks and then mice were i.p injected with Zj7 (50 mg/kg) or vehicle (corn oil) for 2 weeks 90 min before dark cycle. The 6-week-old male *db/db* mice were purchased from the Jackson Laboratory. Mice carrying the homozygous mutation for spontaneous diabetes (*Lepr*^*db*^) show marked obesity at 3–4 weeks of age. For test of Zj7 in *db/db* mice, mice were injected i.p. with Zj7 (50 mg/kg) or vehicle (corn oil) for 16 days. The mice used in the experiments were sacrificed by CO_2_ inhalation. The mice were maintained according to the guidelines of Seoul National University Animal Experiment Ethics Committee. All animal experiments were performed after receiving approval of the Institutional Animal Care and Use Committee of the Institute of Laboratory Animal Resources, Seoul National University (Institutional Animal Care and Use Committee permit number: SNU-170704-7-6). All experiments were carried out in accordance with the ARRIVE guidelines and regulations.

### Elevated plus maze test

The elevated plus maze test was performed after 20 h of vehicle (corn oil) or Zj7 (50 mg/kg) injection in lean mice fed normal chow diet. The elevated plus maze consisted of two black open arms without walls and two black closed arms with walls (30 cm long, 5 cm wide, 40 cm high). Mice were placed in the central quadrant with their backs to an open arm. The ANY-maze video tracking system (Anilab) was used to monitor and analyze the time the mice spent in the open and closed arms and their entries into these arms for 10-min session. Anxiety levels were assessed based on reduced entries into the open arms and reduced time spent in these areas.

### Conditioned taste aversion test

The lean mice fed normal chow diet designated for the conditioned taste aversion (CTA) experiment were water deprived and subjected to a schedule of daily access to water from a bottle for 20 min to establish baseline water consumption. After a 4-day baseline period, the CTA acquisition phase was initiated. On day 5, mice were exposed to a 0.1% sucrose solution for 20 min, and 30 min later they were injected i.p. with vehicle (corn oil) or Zj7 (50 mg/kg). The following day, during the memory retrieval test, all mice were again given access to a 0.1% sucrose solution for 20 min to evaluate the taste aversion. Sucrose consumption during the retrieval test compared to sucrose consumption during the acquisition test was used as a measure of CTA strength.

### Metabolic chamber and body composition measurements

All mice were acclimated to the metabolic cage for two days, and metabolic parameters and locomotor activity were measured for 48 h using Phenomaster (TSE system, Germany). The bar graph represents the average of 48 hourly observations for each of the vehicle-injected and Zj7-injected groups. Body composition was measured using Minispec LF50 (Bruker, Germany) at the Korea Mouse Phenotyping Center (KMPC), Seoul National University.

### Glucose tolerance test (GTT) and insulin tolerance test (ITT)

For GTT, mice were fasted for 16 h during the dark cycle before intraperitoneal injection of D-glucose in distilled water solution (2.5 g/kg). Blood glucose levels were measured from the tail vein at 30, 60, 90 and 120 min after glucose injection. For ITT, mice were fasted for 4 h during the light cycle. Insulin (0.75 U/kg) was intraperitoneally administered. Blood glucose levels were measured at 30, 60, 90 and 120 min after insulin injection.

### RNA-seq experiments

10 weeks old male mice were IP injected with vehicle, Dex (10 mg/kg), or Dex + Zj7 (50 mg/kg) for 24 h. Hypothalamic ARC regions were dissected and total RNA was isolated using TRIzol (Invitrogen). RNA samples were treated with DNase (Invitrogen) and then purified using RNeasy mini kit (Qiagen). The integrity of the RNA was validated by bioanalyzer. RNAseq libraries were generated according to the TruSeq Stranded mRNA sample preparation guide (Part # 15,031,047 Rev. E). The RNAseq libraries were verified by bioanalyzer and real-time RT-PCR. To sequence the RNA-seq libraries, Illumina HiSeq 2000 was used.

### RNA-seq data analysis

Preprocessing for raw reads was performed to remove low-quality reads and artifacts such as adapter sequences, contaminant DNA, and PCR duplicates. The retained reads were mapped against the USCS mouse reference genome (mm10) using Bowtie2 (version 2.3.4.1) and HISAT2 (version 2.1.0) to generate aligned reads^34^. The mapped reads were assembled using the StringTie (version 2.1.3b) to identify potential transcripts^35^. After assembly, quality check and normalization were conducted to minimize systematic bias for statistical analysis. Genes with a count value of 0 in at least one sample were excluded from the analysis. The degree of similarity between samples in each group was examined using Pearson’s coefficient for each sample to confirm the reproducibility of repeated samples. The expression levels of transcripts were estimated from the read count and FPKM (Fragments Per Kilobase of transcript per Million mapped reads) normalized values. The differentially expressed genes (DEGs) with log_2_ fold change (FC) ≥ 1.5 (*p* < 0.05) were determined by edgeR package. The DEGs were then used for downstream analyses including hierarchical clustering analysis and functional analysis. Functional analysis of DEGs was performed using Ingenuity Pathway Analysis (IPA) and DAVID^36^.

### RNA isolation and quantitative RT-PCR

Total RNA was isolated from hypothalamic ARC, and brown adipose tissue with Trizol, and converted to cDNA using SuperScriptIII (Invitrogen). Quantitative RT-PCR was conducted with by SYBR based real-time PCR assay (TOPreal™ qPCR 2 × PreMIX SYBR with high ROX; Enzynomics™). The relative gene expression values were normalized to *Cyclophilin A* by comparative Ct method. Relative expression levels were calculated from three separate experimental replicates. The following primers were used for qPCR: mouse *Agrp*, 5’- CCC AGG TCT AAG TCT GAA TGG C, 5′-TTC TGT GGA TCT AGC ACC TCC G, *Nfkbia*, 5′-AGG ACG AGG AGT ACG AGC AA, 5′-AAG CCA AGT GGA GTG GAG T, *Ptpn11*, 5′-AAG TAC CCG CTG AAC TGT GC, 5′-AAT GCT CCA CCA GGT CTG TC, *Npy*, 5′-ACT CCG CTC TGC GAC ACT ACA T, 5′-GCG TTT TCT GTG CTT TCC TTC A, *Bcl6*, 5′-AGC CGG CTC AAT AAT CTC GT, 5′-TTG CAG AAG AAG GTC CCA TT, *Cdkn1a*, 5′-GTC CAA TCC TGG TGA TGT CC, 5′-CAG GGC AGA GGA AGT ACT GG, *Sgk1*, 5′-ACG CAG CTG AAA TAG CCA GT, 5′-TAC GGC TGC TTA TGG AGG AC, *Sgk3*, 5′-AAT GGA CAG CCC AAG ACA TC, 5′-CAA AGC TGC CCT TTC CAA TA, *Ucp1*, 5′-CAC CTT CCC GCT GGA CAC T, 5′-CCC TAG GAC ACC TTT ATA CCT AAT GG, *Ucp2*, 5′-ACC ATT GCA CGA GAG GAA GG, 5′-TCT TGA CCA CAT CAA CGG GG, *β3-AR*, 5′-GCT CCG TGG CCT CAC GAG AA, 5′-CCC AAC GGC CAG TGG CCA GTC AGC G, *Dio2*, 5′-AAT TAT GCC TCG GAG AAG ACC G, 5′-GGC AGT TGC CTA GTG AAA GGT, *Prdm16*, 5′-TGA GCC CCA AGG AGT CTA TG, 5′-GCG TGG AGA GGA GTG TCT TC and *Cyclophilin A,* 5′-GTC TCC TTC GAG CTG TTT GC, 5′-GAT GCC AGG ACC TGT ATG CT.

### Immunohistochemistry and in situ hybridization assays

Before performing standard perfusion with PBS and 4% PFA, mice were injected with Avertin intraperitoneally, and then fixed in 4% PFA o/n at 4 °C. Immunohistochemistry was carried out by incubating brain slices with antibodies against OTP (Homemade; 1:2000; DOI: https://doi.org/10.1038/s41467-018-04377-4), POMC (H-029-30; Phoenix Pharmaceutical, 1:2000) and TH (ab-152; Merk Millipore, 1:2000) in blocking buffer o/n at 4 °C. Slides were washed with PBST and incubated with secondary antibodies conjugated with fluorescence followed by staining with DAPI. Brown adipose tissue for IHC was fixed in 10% formalin and embedded in paraffin. Paraffin blocks were sectioned using a microtome at a thickness of 5 µm per section. After paraffin was removed with xylene, IHC was performed by incubating brown adipose tissue sections with antibodies against UCP1 (UCP11-A, Alpha Diagnostics, 1:1000) and TH (ab-152; Merk Millipore, 1:2000) in blocking buffer o/n at 4 °C. Slides were washed with PBST and incubated with fluorescence-conjugated secondary antibodies followed by DAPI staining. In situ hybridization was performed according to the protocol. Basically, indicated RNA probes were incubated at 68 °C overnight. After hybridization, slides were incubated in washing buffer (50% formamide, 1 × SSC solution and 0.1% Tween20) for 1 h, blocked in MABT buffer + 4% BSA for 1 h, and incubated with an anti-digoxigenin-AP antibody (11093274910 Roche, 1:5000) in MABT buffer + 2% BSA. The next day, the color reaction was performed with NBP/BCIP after washing with MABT buffer.

### In vivo* GST pull-down, co-immunoprecipitation and immunoblotting*

Mouse Bsx was cloned to GST-tagged pEBG vector and rat glucocorticoid receptor full length was cloned to myc-tagged pCS2 vector. Plasmids were transfected to HEK293T cells by calcium phosphate method. Cells were lysed in tissue lysis buffer (200 mM NaF, 20 mM Na-pyroPO4, 200 mM Tris–HCL, pH 8.0; 150 mM NaCl; 4 mM Na_3_VO_4_; 1 mM EDTA, pH8.0; 0.5% NP-40; 10% Glycerol; 2 mM PMSF) and the lysate was subjected to GST pulled down using Glutathione Sepharose 4B (#17075601; GE Healthcare Life Science) by overnight incubation at 4 °C. 10-week-old male mice were injected with either vehicle, Dex (10 mg/kg), or Dex (10 mg/kg) + Zj7 (50 mg/kg) for 2 h and then dissected hypothalamic tissues were lysed in lysis buffer, followed by immunoprecipitation with anti-Bsx antibody. Beads were then dissolved in SDS loading buffer by boiling at 100 °C for five minutes and proteins were separated on sodium dodecyl sulfate poly acrylamide (SDS-PAGE) gels, and transferred to nitrocellulose blotting membrane (Amersham™ Protran™ 02. μm NC, #10600001; GE Healthcare Life science). The membranes were blocked and incubated with primary antibodies against myc (Millipore; 1:5000), GST (sc-138; Santa cruz, 1:5000), GR (Santa Cruz; 1:1000), and Bsx (gift from Dr. Jae W. Lee, 1:5000) o/n. Next day, the membranes were incubated with secondary antibodies, and developed using ECL solution (Clarity Max western ECL substrate, #170562; BIO-RAD).

### Chromatin immunoprecipitation assay

ChIP assays with mHypoA-2/12 cells were performed as described^37^. mHypoA-2/12 cells were maintained in DMEM media with 10% FBS. For ChIP, cells were seeded in 10-cm dishes, treated with vehicle, Dex (0.1 μM) and Zj7 (50 μM) for 2 h before harvest. The antibodies used for ChIP assays were rabbit α-IgG antibody (Santa Cruz) and α-GR antibody (Santa Cruz). The following primers were used for ChIP-PCR; *Agrp*-GRE, forward: 5′-GGC AGA GAC CTG ACA ACA CA, reverse: 5′-ACG CCC CCT TTC TAC CTA AG; *Per1*-GRE, forward: 5′-CTC GGG AGT CCA TGG GTT T, reverse: 5′-CTA GCG CTG AGA ACA GGA TGT TC; *Untr6*, forward: 5′-TCA GGC ATG AAC CAC CAT AC, reverse, 5′-AAC ATC CAC ACG TCC AGT GA.

### Statistical analysis

Data are presented as means ± s.e.m. Statistical differences were determined by Student t test, one-way analysis of variance (ANOVA) followed by Tukey’s test and two-way analysis of variance (ANOVA) followed by Bonferroni’s post hoc test using Prism 9 (GraphPad Software, La Jolla, CA) software. Statistical significance is expressed based on *p < 0.05, **p < 0.01, ***p < 0.001, ****p < 0.0001, and ns is not significant (*p* > 0.05).

### Supplementary Information

Supplementary Figures.

## Data Availability

Datasets analyzed during the current research will be available from corresponding author on reasonable request. RNA sequencing data used in this study is available in the Gene Expression Omnibus database (https://www.ncbi.nlm.nih.gov/geo/; accession number: GSE260739).
