# Supplementary material for: Characterization of a new selective glucocorticoid receptor modulator with anorexigenic activity
Source: Sci Rep. 2024 Apr 3;14:7844. doi: 10.1038/s41598-024-58546-1 (PMC10991430; doi:10.1038/s41598-024-58546-1)
Supplement: Supplementary file 1 — Supplementary Figures. [file 41598_2024_58546_MOESM1_ESM.pdf]

Supplementary information

Characterization of a new selective glucocorticoid receptor modulator with anorexigenic activity

Junekyoung Lee[1#], Yeonghun Song[1#], Young A. Kim[1], Intae Kim[1], Jooseon Cha[1], Su Won Lee[1], Yoonae Ko[1], Chong-Su Kim[2], Sanghee Kim[1]* and Seunghee Lee[1]*

[1]College of Pharmacy and Research Institute of Pharmaceutical Sciences, Seoul National University, Seoul 08826, Korea, [2]Department of Food and Nutrition, College of Natural Information Sciences, Dongduk Women's University, Seoul 02748, Korea

```
    ***           *****
CCAGGGAACAGTTCGTTCTGTTTC  Agrp-GRE
CTAGGGAACATCGTGTTCTCTTGG  Per1-GRE
CCTGGGAACATACTGTGCCCGCCA  Cdkn1a-GRE

    GGTACANNNTGTTCT       consensus GRE
TTTGGTTACAAACTGTTCTTAAAA  MMTV-GRE
TAACAGGACAGGCTGTCCTCGCAG  MT1-GRE
```

Supplementary Figure S1. Sequences of *Agrp*-GRE, *Per1*-GRE, and *Cdkn1a*-GRE as well as other conventional GREs including *MMTV*-GRE and *MT1*-GRE. The unique sequences in *Agrp*-GRE and *Agrp*-GRE-like motifs are indicated with an asterisk.

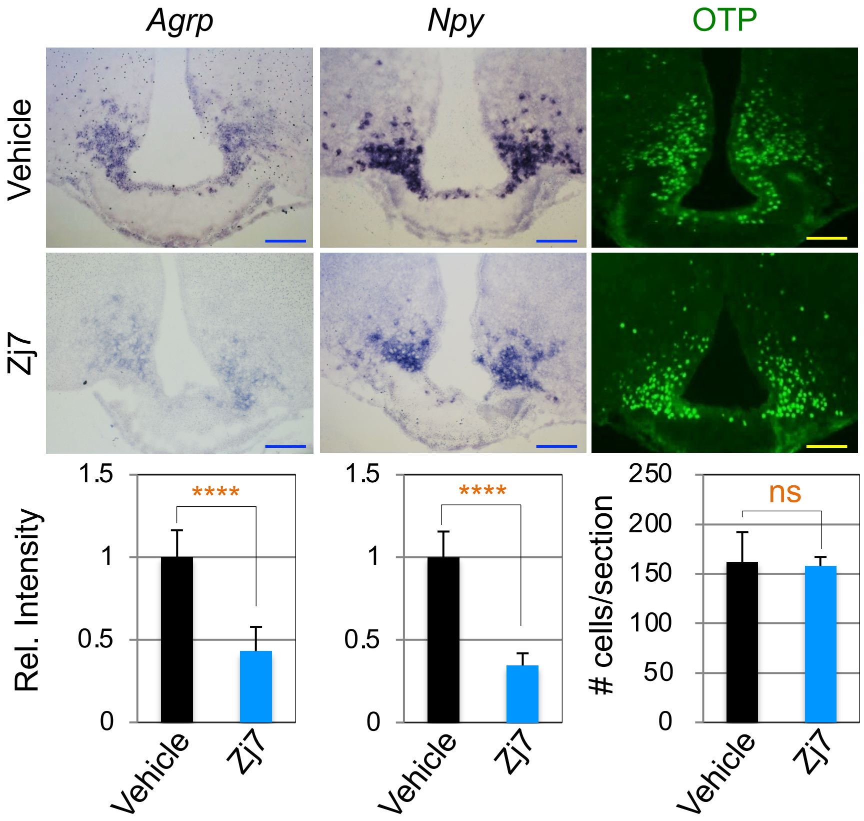

Supplementary Figure S2. *In vivo* evidence of Zj7 in suppressing fasting-induced AgRP expression. In situ hybridizations for *Agrp* and *Npy* were performed with serial sections of the ARC region of lean mice (n=4 in each group) intraperitoneally injected with either vehicle or Zj7 (50 mg/kg) for 12 hours during fasting. Immunohistochemistry assay with anti-OTP antibody labeled AgRP neurons and showed no neuronal cell fate change or cell death by Zj7. Representative images are shown. Quantifications were done by the relative intensity of signals or counting the number of labeled cells using ImageJ in three rostral to caudal sections for each mouse. Data are mean ± SD. Statistical differences were determined by Student's t-test; ****p < 0.0001, and 'ns' indicates not significant. Scale bars: 100 mm.

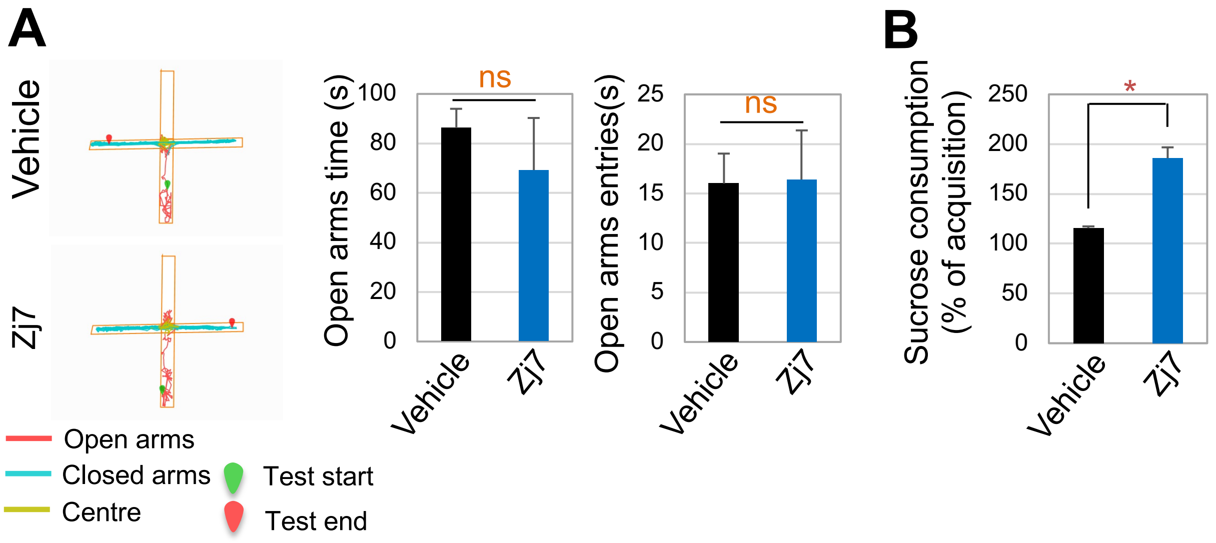

Supplementary Figure S3. (A) Elevated Plus Maze (EPM) test. The EPM test was performed. Lean mice (n=5 in each group) were intraperitoneally injected with either vehicle or Zj7 (50 mg/kg). The elevated plus maze made of plastic and consisted of two black open arms without walls and two black enclosed arms with walls (30-cm long, 5-cm wide, 40-cm high). The maze was placed 60 cm above the floor and mice were introduced into the center quadrant with their back facing an open arm. The ANY-maze video tracking system (Anilab) was used to track and analyze the time of mice spent in the open arms and their entries into the open arms throughout a 10-min session. Anxiety was evaluated by fewer movements into the open arms and less time spent there. (B) Conditioned Taste Aversion (CTA) test. Lean mice were injected with vehicle or Zj7 (50mg/kg) after the first 0.1% sucrose consumption on the acquisition test day. The initial water consumption baseline of each animal was similar. The percentage of sucrose consumption on the retrieval test day was increased in the Zj7-injected lean mouse group (n=6) compared to the vehicle-injected group (n=6). Data are mean ± SD. Statistical differences were determined by Student's t-test; $*p < 0.05$, and 'ns' indicates not significant.

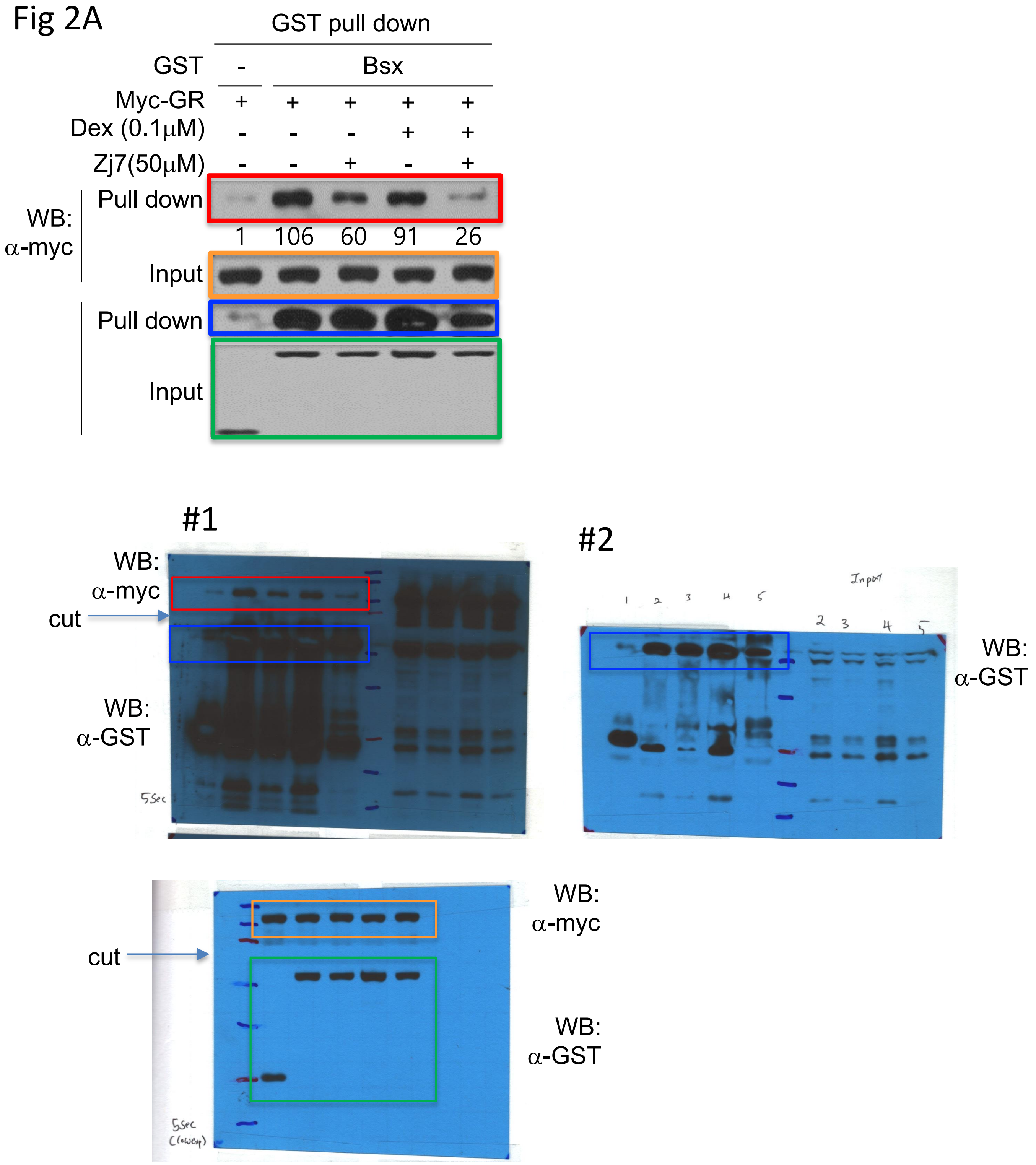

Supplementary Figure S4. Full-length blots of GST-Bsx and Myc-GR in the GST pull-down assay (shown as cropped images in Figure 2A). The nitrocellulose membrane was cut into a small piece after gel transfer for myc-GR and GST-Bsx. Although the membrane was exposed to the film for only 5 seconds, the GST signal was too strong in the pull-down sample (#1 blot), so we reprobed only the lower part of the nitrocellulose membrane and performed a Western blot with anti-GST antibody to get the clear GST signal in the pull-down samples (#2 blot)

Fig 2B

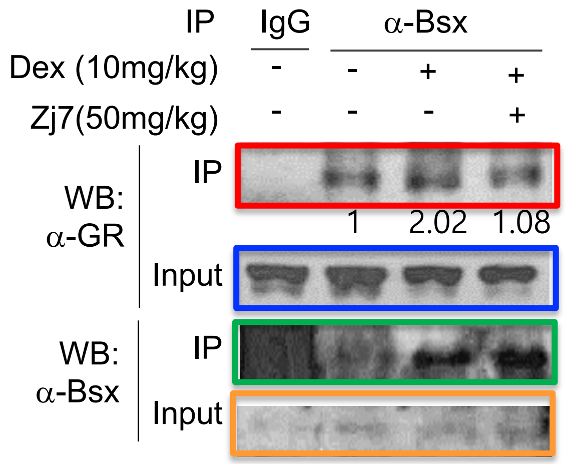

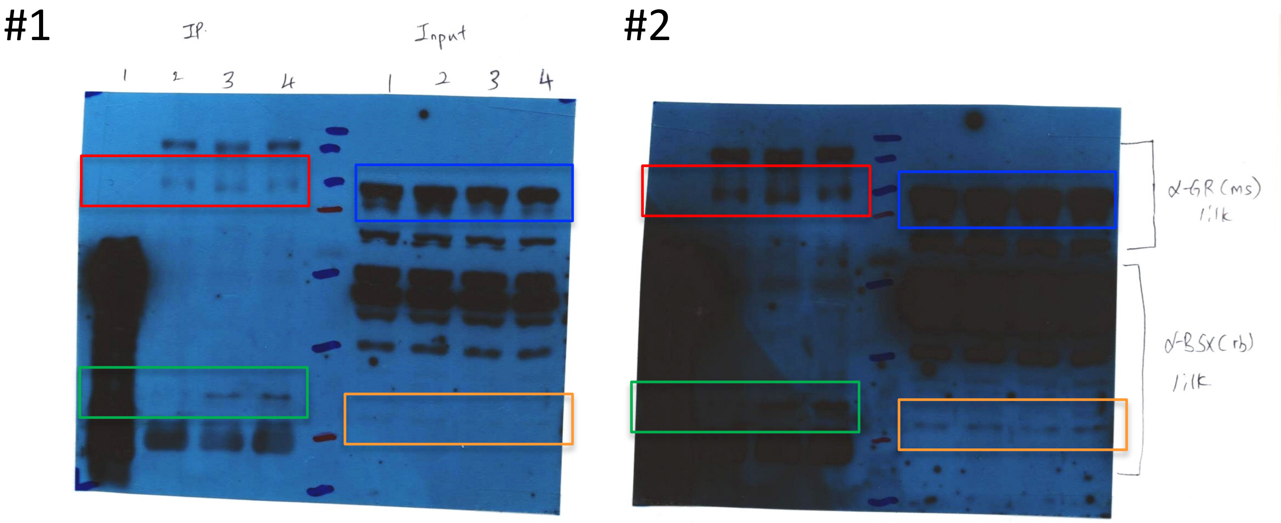

Supplementary Figure S5. Full-length blots of coimmunoprecipitation of GR and Bsx using mouse hypothalamus tissue (shown as cropped images in Figure 2B). After gel transfer, the nitrocellulose membrane was cut into a small piece for GR and Bsx. Blot #1 was exposed to the film for 3 minutes and blot #2 for 7 minutes.
